# Common Features of Environmental *Mycobacterium chelonae* from Colorado Using Partial and Whole Genomic Sequence Analyses

**DOI:** 10.1007/s00284-023-03589-2

**Published:** 2024-01-18

**Authors:** Kayden G. Glauser, Reagan E. Kelley, William J. Leonard, Jo Hendrix, Suzanne Petri, Eric I. Tong, Yvonne L. Chan, Ettie M. Lipner, Stephanie N. Dawrs, Jennifer R. Honda

**Affiliations:** 1 Department of Science, Principles of Experimental Design in Biotechnology, Rock Canyon High School, Littleton, CO 80124 USA; 2 Computational Bioscience Program, University of Colorado Anschutz Medical Campus, Aurora, CO 80045 USA; 3 Aina Informatics Network, ‘Iolani School, Honolulu, HI 96826 USA; 4 National Institute of Allergy and Infectious Diseases, National Institutes of Health, Bethesda, MD 20892 USA; 5 Center for Genes, Environment and Health, National Jewish Health, Denver, CO 80206 USA; 6 Department of Cellular and Molecular Biology, School of Medicine, University of Texas at Tyler Health Science Center, Tyler, TX 75708 USA

## Abstract

Nontuberculous mycobacteria (NTM) are environmentally acquired opportunistic pathogens that cause chronic lung disease in susceptible individuals. While presumed to be ubiquitous in built and natural environments, NTM environmental studies are limited. While environmental sampling campaigns have been performed in geographic areas of high NTM disease burden, NTM species diversity is less defined among areas of lower disease burden like Colorado. In Colorado, metals such as molybdenum have been correlated with increased risk for NTM infection, yet environmental NTM species diversity has not yet been widely studied. Based on prior regression modeling, three areas of predicted high, moderate, and low NTM risk were identified for environmental sampling in Colorado. Ice, plumbing biofilms, and sink tap water samples were collected from publicly accessible freshwater sources. All samples were microbiologically cultured and NTM were identified using partial *rpoB* gene sequencing. From these samples, areas of moderate risk were more likely to be NTM positive. NTM recovery from ice was more common than recovery from plumbing biofilms or tap water. Overall, nine different NTM species were identified, including clinically important *Mycobacterium chelonae*. MinION technology was used to whole genome sequence and compare mutational differences between six *M. chelonae* genomes, representing three environmental isolates from this study and three other *M. chelonae* isolates from other sources. Drug resistance genes and prophages were common findings among environmentally derived *M. chelonae,* promoting the need for expanded environmental sampling campaigns to improve our current understanding of NTM species abundance while opening new avenues for improved targeted drug therapies.

**Supplementary Information:**

The online version contains supplementary material available at 10.1007/s00284-023-03589-2.

## Introduction

Nontuberculous mycobacteria (NTM) are opportunistic pathogens of the Genus *Mycobacteria* in the Family *Mycobacteriaceae*. Within this genus, there are more than 195 identified species, consisting of both tuberculous and nontuberculous species [1]. Infections caused by *Mycobacterium tuberculosis* can lead to tuberculosis, whereas NTM species can cause lung disease (LD) and other illnesses, including infections of the lymph nodes, bone, and skin [2]. Species of NTM that cause LD include the *Mycobacterium avium* complex (MAC) and *Mycobacterium abscessus,* while skin infections are often caused by *Mycobacterium chelonae* [3]. It is postulated that NTM infections are contracted through extended exposure to the natural environment and municipal water sources, such as in household plumbing sources that contain NTM [4]. Water pipes are a hot spot niche for NTM, as they provide the bacteria with a surface to adhere with ample space to grow [5]. Furthermore, NTM are resilient against environmental fluxes and can thrive in conditions where other bacteria cannot, including low oxygen, absence of moisture, high temperatures such as hot water heating systems of up to 50 °C, and chlorination [5–7].

Geographic areas of the United States have been identified as hot spots for NTM infection by epidemiological studies using spatial and cluster analyses, patient population-based data [8, 9], and nested case–control studies [10]. However, the corresponding microbiological studies to confirm the presence of NTM in those environments are comparably less in number. One U.S. hot spot for which both clinical and environmental studies have been performed is Hawai’i [11–13] and accompanying studies have shown that features [14–16] such as metals, including vanadium in groundwater aquifers [17], influence NTM presence and diversity in Hawai’i. Further analysis proposed various human exposure pathways through soil, streams, and groundwater [14–16]. A study by Winthrop et al*.,* reported that in 2008 and 2015, the prevalence of NTM LD in Hawai’i was consistently in the high range with more than 17/100,000 person years, whereas continental, mid-west states like Colorado showed lower rates of 5–7/100,000 person years [18].

Ecological epidemiological NTM studies were performed in Colorado using patient data from electronic medical records and ZIP code-level sociodemographic and environmental exposure data from the U.S. Geological Survey, Department of Agriculture and the Census Bureau to identify low, moderate, and high-risk clusters of NTM LD [19]. Healthcare associated transmission of NTM among people with cystic fibrosis (CF) has been investigated at a Colorado Adult CF program, finding low risk of NTM transmission [20]. But certain environmental conditions, such as the presence of molybdenum in Colorado watersheds was associated with increased NTM disease risk [21]. Using molybdenum concentrations in watersheds across Colorado, regions of low, moderate, and high NTM LD risk were identified [21]. However, unlike Hawai’i, microbiological studies to probe for NTM in non-healthcare associated Colorado environments has not been performed.

Importantly, NTM LD continues to pose a substantial financial burden on the country. For the 2010 year, it was estimated that $815 million of direct care costs (76% of these costs were related to medications) was used to treat 86,244 national NTM patient cases, of which 87% comprised inpatient treatment costs [22]. NTM was recently noted as the leading cause of drinking water associated illness, emergency room visits, hospitalizations, and death [23]. Thus, gaining a broader understanding of the geographic diversity of NTM may help to define hot spot areas to design novel strategies to reduce disease burden.

In this study, we used a combination of microbiological culture and genetic sequencing methods to isolate and identify NTM from environmental samples collected from low, moderate, and high predicted NTM LD risk regions in Colorado based on Lipner et al. 2020. From these samples, viable *Mycobacterium chelonae* was commonly recovered*.* Because *M. chelonae* was the most clinically relevant NTM species identified from the Colorado environmental samples studied, we also performed MinION whole genome sequencing to compare genetic differences between different environmental isolates of *M. chelonae*. We found little differences in species diversity, richness, and core genomes demonstrating the universality of *M. chelonae*.

## Materials and Methods

### Environmental Sampling

The selection of sampling locations was guided by the geospatial modeling conducted by Lipner et al. 2020 that predicted three geographic areas of LD risk in Colorado –low, moderate, and high based on environmental molybdenum levels [21]. Within each of these three risk areas, two unique publicly accessible sites, *e.g*., gas stations, were sampled per risk area, *i.e.,* Aspen/Vail and Eagle as low risk areas, Dillon and Boulder as moderate risk areas, and Ten Mile Creek and Denver as high-risk areas (Online Resource 1). Within these six geographic locations, three different locations were sampled per site and three different types of samples were collected including: (1) ice from public ice machines, (2) plumbing biofilms from public bathroom sinks, and (3) 2 L of public tap water samples from which plumbing biofilms were collected. Sampling was performed between October and December 2021. Thus, a total of 54 environmental samples were collected for this study.

Ice samples were obtained by completely filling two, 1-gallon sterile bags with ice from ice machines located within each sampling site. Sterile synthetic flock dual-tipped swab applicators (Puritan #25-3306 2HBT, Guilford, ME) were used to collect plumbing biofilms from public bathroom sinks. Swabs were used to thoroughly wipe the underside of bathroom sink spouts ten times using back and forth and circular motions. Tap water samples were collected in two sterilized 1 L glass Pyrex bottles from the same sink where the plumbing biofilm swab was sampled. Ice and swabs were stored at − 20 °C and water was stored at 4 °C until they were cultured.

### Microbiological Culturing

Ice was melted to water at room temperature and water samples were brought to room temperature before culturing. To culture NTM from melted ice and tap water, 1.2 L of each sample was vacuum filtered through sterile 0.2 μm filters (Millipore, MA). Filters were cut out of the filtering apparatus using sterilized forceps and razor blades. Once removed from the filter apparatus, filters were further cut into 12–16 equally sized pieces. Using sterile forceps, filter pieces were transferred into sterile flasks containing 10 mL of autoclaved MilliQ water. Sample flasks were vortexed on high for 30 s. To remove microorganisms other than NTM, 450 µL of sample solution was transferred to sterile 1.5 mL microcentrifuge tubes to which 50 µL of 1% cetylpyridinium chloride (CPC) was added. Tubes were vigorously vortexed and incubated for 30 min at room temperature. Each disinfected sample was vortexed again and 100 µL was spread onto two Middlebrook 7H10 plates containing oleic acid-dextrose-catalase (OADC) enrichment.

To process water biofilm samples, swabs were transferred into sterile 5 mL screw cap tubes containing 2 mL of sterile MilliQ water. To dislodge microorganisms from the swab, sample tubes were vortexed for 1 min on high. Samples were disinfected with 1% CPC for 30 min and spread plated on 7H10-OADC agar plates following the same procedures as the water and ice samples described above. Culture plates were sealed in polybags and incubated at either 30 °C or 37 °C for 21 days. Following incubation, culture plates were examined for microbial growth. Colonies that appeared to be NTM based on morphology were picked and inoculated into Middlebrook 7H9 broth supplemented with albumin-dextrose-catalase (ADC) enrichment. Once bacterial cultures were turbid, 1 mL of each culture was transferred into sterile 1.5 mL tubes and centrifuged at 13,000 × *g* for 2 min to pellet bacteria. Supernatants were discarded, and pellets were stored at − 80 °C until DNA extraction.

### DNA Extraction, Sanger Sequencing, and NTM Identification

To extract DNA from bacterial pellets, the protocol by Epperson et al. was used [24]. Pellets were brought to room temperature and suspended in 100 µL of lysis buffer with 25 µL of lysozyme (100 mg/ml). Samples were incubated overnight at 37 °C. After overnight incubation, 25 µL of proteinase K (2.5 mg/ml) and 50 µL of 20% sodium dodecyl sulfate (SDS) were added, and the samples were incubated for an additional hour at 37 °C. Each sample was transferred to a 96 deep-well plate containing 0.1 mm zirconium beads (BioSpec, OK) and binding buffer from ZR-96 Genomic DNA Clean & Concentrator Kit (Zymo Research, CA). The sample plate was shaken for three minutes at 30 cycles/second on the Tissuelyser (Qiagen, MD), then centrifuged at 14,000×*g* for 2 min. Sample supernatants were transferred to a Zymoclean 96 well column plate and the remainder of the procedure followed kit instructions (Zymo Research, ZR-96 Clean & Concentrator-5, Catalog # D4067).

To identify NTM, PCR reactions were set up with sample DNA for Sanger sequencing of a 711 bp region of the RNA polymerase beta subunit (*rpoB*) gene at QuintaraBio (CA, USA) [25]. Sequencing results were trimmed for quality, then imported into the BLAST algorithm of the National Center for Biotechnology Information (NCBI) GENBank system to compare to strain references.

### MinION Whole Genome Sequencing and Analyses

Six *M. chelonae* isolates identified by *rpoB* sequencing were selected for MinION sequencing using Oxford Nanopore Technologies (ONT) **(**Table 2**).** Due to the high DNA concentration requirements of MinION, the six NTM isolates were regrown in 50 mL 7H9 liquid cultures. Once cultures were turbid, the entire culture was centrifuged at 13,000×*g* for 2 min to create large pellets. DNA was extracted from pellets following the procedures described above and reagent volumes were doubled to account for increased bacterial pellet size.

DNA libraries were prepared using 400 ng of each *M. chelonae* DNA sample and multiplexed with the Rapid Barcoding kit (SQK-RBK004, ONT) according to manufacturer’s instructions. The multiplexed DNA libraries were cleaned and concentrated with AMPure XP beads (Agencourt) following manufacturer’s instructions. The multiplexed DNA libraries were loaded onto the R9.4.1 Flowcell (ONT FLO-MIN106D) and sequenced on a MinION instrument for 48 h.

ONT reads were base called and demultiplexed using Guppy v6.2.1 [26]. Reads were assembled using Canu v2.2 [27] and assemblies were annotated using Prokka v1.13 [28]. Species identity was confirmed by aligning the annotated 16S rRNA and *rpoB* genes to the NCBI database (accessed June 26, 2023) with BLAST. The pangenome was profiled using Roary [29]. Contigs shorter than 200 kb were aligned to the NCBI database (accessed June 11, 2023) with BLAST and plasmid identity was determined using PlasIDome at https://github.com/jrhendrix/plasIDome.

Phylogenetic relatedness was determined through average nucleotide identities (ANIs) of the full genomes using OrthoANI [30] and was visualized using a custom script in R. Because little is known about integrated viral genomes among the *M. chelonae*, integrated prophages were detected using Depht on sensitive mode [31]. Previously described prophages with sequence similarity to detected prophages were identified from the ‘Mycobacterium prophages – Sep 2021’ database (accessed September 27, 2023) using phagesDB (https://phagesdb.org/blast/) and verified using BLAST. Data generated in this study were deposited in GenBank as BioSamples SAMN37846682-SAMN37846688, under BioProject ID PRJNA1028592.

### Statistical Analyses

Statistical testing was performed in Prism 9.5.1. The Chi-squared test was used to compare NTM presence per risk area and in ice/plumbing biofilms/tap water due to the small sample sizes.

## Results

Results are summarized in Table 1. From the 54 total environmental samples collected, 22 (41%) were NTM positive. Because our sampling strategy was based on epidemiological modeling of NTM LD prevalence rates [21], we hypothesized that NTM are less frequently recovered from low-risk areas compared to high-risk areas. The two lowest NTM culture positivity rates corresponded to the geographic areas of low risk, Aspen/Vail and Eagle, with 2/9 samples per area (22%) being NTM positive. Among the two moderate NTM LD risk areas sampled, Dillon showed the highest NTM positivity rate among all six sites, (9/9, 100%), whereas 4/9 samples from Boulder were NTM positive (44%). Then among the two high risk areas sampled, Denver showed a higher NTM positivity rate (4/9, 44%) compared to the second high risk area, Ten Mile Creek (1/0, 11%). Overall, areas of moderate risk were significantly more likely to be NTM positive than the low and high-risk areas (**, p = 0.0037).

**Table 1:** NTM culture positivity rates from Colorado environmental samples

NTM Risk Region:	City:	Ice positivity:	Plumbing biofilm positivity:	Tap water positivity:	Overall positivity:
Low	Aspen/Vail	66.7% (2/3)	0% (0/3)	0% (0/3)	22.2% (2/9)
Low	Eagle	33.3% (1/3)	33.3% (1/3)	0% (0/3)	22.2% (2/9)
Moderate	Boulder	66.7% (2/3)	0% (0/3)	66.7% (2/3)	44.4% (4/9)
Moderate	Dillon	100% (3/3)	100% (3/3)	100% (3/3)	100% (9/9)
High	Denver	100% (3/3)	0% (0/3)	33.3% (1/3)	44.4% (4/9)
High	Ten Mile	33.3% (1/3)	0% (0/3)	0% (0/3)	11.1% (1/9)
All	All Cities	66.7% (12/18)	22.2% (4/18)	33.3% (6/18)	40.7% (22/54)

**Fig. 1:**
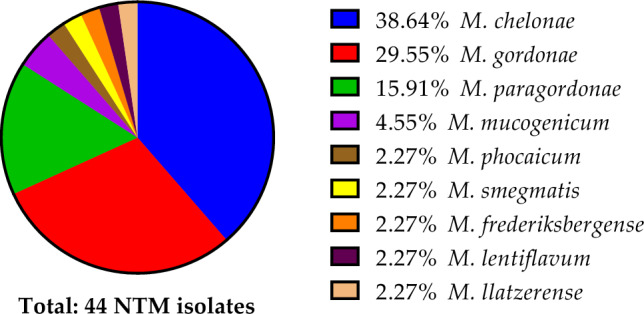
Overall species diversity observed within Colorado environmental samples from 44 recovered NTM isolates

When analyzed by sample type, NTM recovery from ice was significantly more common than recovery from plumbing biofilms or tap water **(**Table 1**)** (*, p = 0.0185)**.** In total, nine NTM species were identified from 44 isolates in which *M. chelonae* was the most frequently isolated (39%, n = 17), followed by *Mycobacterium gordonae* (30%, n = 13), *Mycobacterium paragordonae* (16%, n = 7), *Mycobacterium mucogenicum* (5%, n = 2), *Mycobacterium phocaicum* (2%, n = 1), *Mycobacterium smegmatis* (2%, n = 1), *Mycobacterium frederiksbergense* (2%, n = 1), *Mycobacterium lentiflavum* (2%, n = 1), and *Mycobacterium llatzerense* (2%, n = 1) (Fig. 1). There were instances where more than one isolate was recovered from a single sample.

**Fig. 2:**
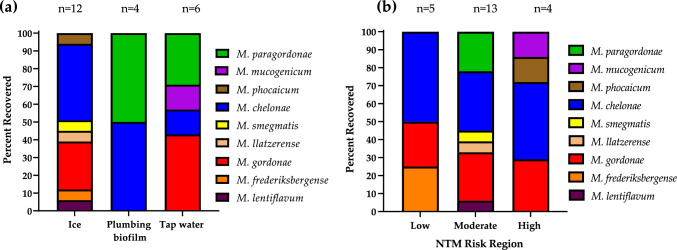
NTM species diversity based on *rpoB* sequencing identification and stratified by **a** Type of sample collected. “n” identifies the number of each particular sample that was NTM positive and the diversity of species found in that sample type. For example, there were 12 NTM positive ice machines harboring seven different species (seven different colored bars) in varying proportions. **b** NTM species diversity modeled by risk area for Colorado NTM LD. “n” identifies the number of different NTM positive locations sampled per risk region

*M. chelonae* was represented in all three different types of samples studied (Fig. 2a), whereas *M. gordonae* was only identified in ice and tap water samples and absent from plumbing samples. *M. paragordonae* was only identified from tap water and plumbing biofilm samples, while *M. phocaicum, M. smegmatis, M*. *llatzerense, M. frederiksbergense,* and *M. lentiflavum* were only detected in ice.

As mentioned, NTM were more frequently recovered from moderate LD risk areas compared to low and high-risk areas (Table 1), which also showed the most diverse spectrum of species, *i.e*., six different NTM species (Fig. 2b). In comparison, three species were recovered from the low-risk area samples and four different species were recovered from the high-risk area samples (Fig. 2b).

**Fig. 3:**
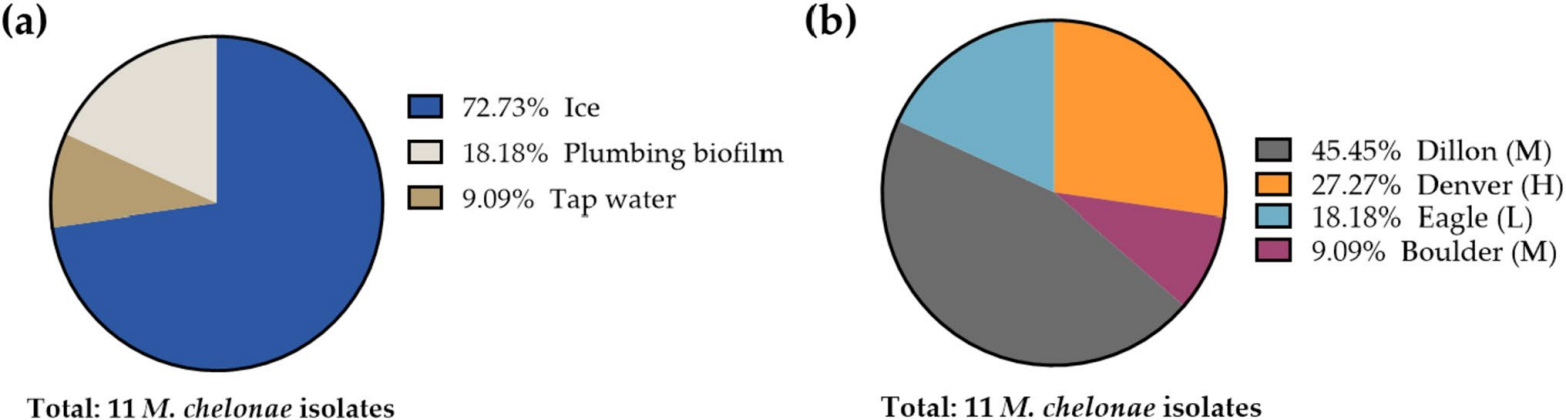
Attributes of the 11 Colorado *M. chelonae* isolates isolated from the same number of unique sites. **a** The proportion of environmental sample types from which *M. chelonae* was microbiologically recovered. **b** Colorado cities from which the most *M. chelonae* was recovered; *M* = moderate risk area, *H* = high risk area, *L* = low risk area

Next, we performed additional studies focused on *M. chelonae* because it was the most frequently recovered NTM species among the Colorado samples, identified in all three regions, and is considered one of the most pathogenic of the rapid-growing mycobacteria. Of the 17 total isolates of *M. chelonae*, 11 represented unique sampling sites. Of the 11 isolates, 73% (n = 8) were cultured from ice, 18% (n = 2) from plumbing biofilms, and 9% (n = 1) were recovered from tap water samples (Fig. 3a). Of the cities that *M. chelonae* was identified, 45% were from Dillon, 27% from Denver, 18% Eagle, and 9% from Boulder (Fig. 3b).

**Fig. 4:**
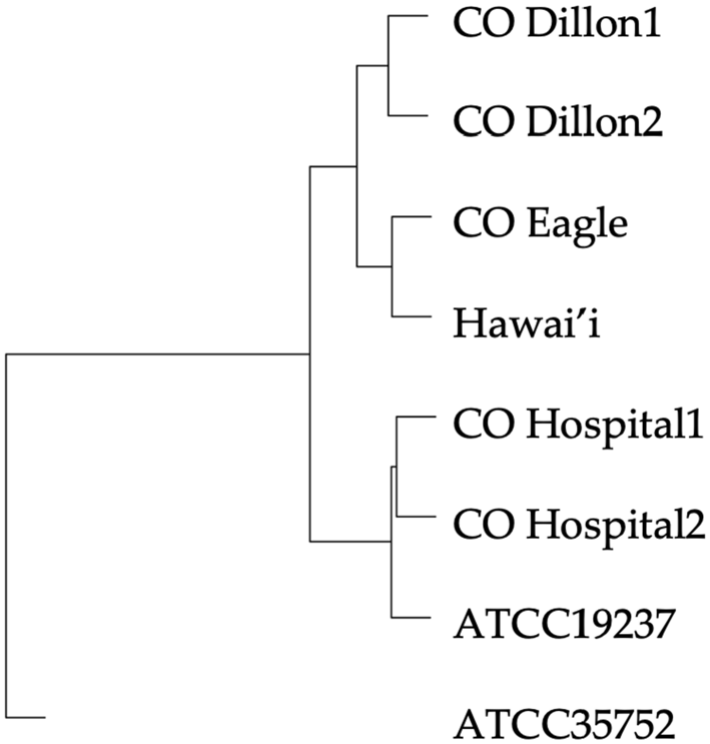
Phylogenomic tree of MinION whole genome sequenced *M. chelonae* isolates from Colorado and a Hawai'i environmental sample

To compare the genetic differences among environmental *M. chelonae* isolates, we performed whole genome sequencing (WGS) using the MinION platform on six *M. chelonae* isolates including three plumbing biofilm isolates from this study and three isolates from separate studies including two environmental Colorado hospital isolates and one environmental *M. chelonae* isolate from Hawai’i (Table 2). We phylogenomically compared the *M. chelonae* isolates by average nucleotide identity (ANI). All six were genetically similar as well as to *M. chelonae* ATCC 19237, with less similarity to *M. chelonae* ATCC 35752. The sample from Eagle, Colorado was most genetically similar to the environmental *M. chelonae* isolate from Hawai’i (99.6% ANI) and they clustered together with the samples collected from Dillon, Colorado (CO Dillion1 and CO Dillion2) (Fig. 4).

**Table 2:** Whole genome sequenced *M. chelonae* isolates and their ID used in Fig. 4

Isolate	Location	Sample source	Plasmid	Plasmid species
CO Dillion 1	Dillon	Plumbing biofilm	No	NA
CO Dillion 2	Dillon	Plumbing biofilm	No	NA
CO Eagle	Eagle	Plumbing biofilm	Yes	*Mycobacterium abscessus*
CO Hospital 1	Denver	Hospital coffee machine	No	NA
CO Hospital 2	Denver	Hospital coffee machine	No	NA
Hawai’i	O’ahu	Kitchen sink faucet	No	NA

Pangenomic analysis revealed that the six assembled genomes and two references genomes of *M. chelonae* contained the genes *modA* and *modC* involved in molybdenum import into the cell and, except for ATCC 35752, the gene *mopII*. Each of the genomes also harbored genes involved in resistance to bicyclomycin (*bcr*), bleomycin (*ble*), daunorubicin/doxorubicin (*drrA*), tetracycline (*tetA*), and beta-lactams (*ampC*). Unlike the two reference *M. chelonae* genomes which were both collected from animal sources, the six sequenced environmental *M. chelonae* isolates contained genes involved in acidic pH tolerance (*adhD*) and in metabolizing terpene, an aromatic hydrocarbon produced by plants (*linC*). Of the six sequenced isolates, only the Colorado samples had genes for an adenylosuccinate synthetase (*purA*) and bile acid-coenzyme A ligase (*baiB*), while only the Hawai’i isolate harbored genes for the DpnIIA methylase (*dpnM*) and the uncharacterized protein YqbO (*yqbO*).

Plasmids and prophages are vectors for horizontal gene transfer of antibiotic resistance and virulence genes within and across NTM species. Genome assemblies were first screened for the presence of plasmids, but only the *M. chelonae* isolate from Eagle, Colorado harbored a plasmid (Table 2). This plasmid was 49,797 bp in length and had over 99% sequence similarity to plasmids pGD69B-1 and pGD42-1 in *M. abscessus*. *M. chelonae* isolates did not show evidence of a recently described plasmid-mediated *erm*(55) gene [32].

**Fig. 5:**
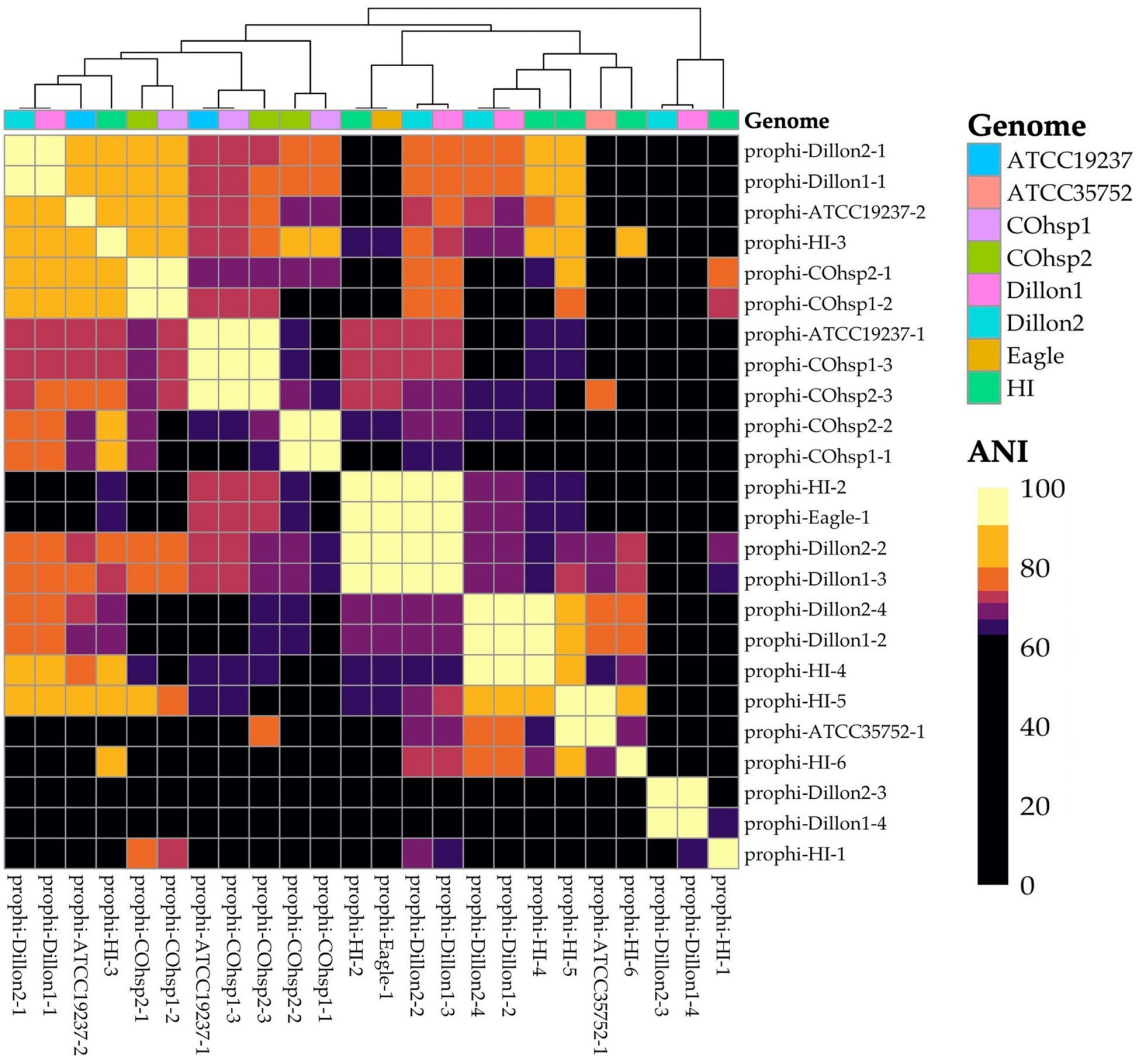
Similarity matrix of identified prophages. Higher ANI (lighter coloration) indicates more sequence similarity

Finally, we probed for integrated prophages, the remnants of past prophage infection, among the *M. chelonae* because very little is known about the roles of prophages in mycobacterial drug resistance. Prophage detection identified 24 unique prophages among the six WGS *M. chelonae* isolates. The two isolates from Dillion contained nearly identical copies of the same four prophages. Of these, two phages were also present in the environmental *M. chelonae* isolate from Hawai’i (prophi-HI-4 and prophi-HI-2) and one other was found in the Eagle isolate (prophi-Eagle-1). The *M. chelonae* isolate from Hawai’i also had four additional phages but only one (prophi-HI-5) demonstrated significant sequence similarity to a prophage in tortoise derived *M. chelonae* ATCC 35752 (prophi-ATCC35752-1; 92% ANI). Nearly identical copies of another three prophages (prophi-COhsp1-1, 2, and 3) were detected in the two *M. chelonae* isolates from Colorado hospital environments (Fig. 5).

## Discussion

Environmental NTM studies that take place in U.S. geographic areas with lower prevalence of LD, such as Colorado, will provide crucial insight to understanding NTM in the environment across different landscapes and how geographical differences can translate to differential human exposures that could lead to infection. Our research aimed to identify environmental NTM from various regions in Colorado, a state with little to no environmental NTM culture data. This study was designed to collect samples from areas predicted to be low, moderate, and high NTM LD risk based on environmental epidemiologic regression models. Within Colorado, NTM positivity and species diversity was highest in the moderate region, specifically in the city of Dillon located in the Blue Colorado watershed, a rural area with sparely distributed populated regions living at high elevation [19].

In a previous Colorado study, the Blue, Upper South Platte, and Middle South Platte Cherry Creek watersheds were associated with increased NTM infection risk [19]. The Dillon Reservoir, located in Dillon connects to the Upper South Platte and Middle South Platte Cherry Creek watersheds. It is therefore possible that the increase in NTM positivity observed in Dillon may be influenced by the local geology and nearby water sources. It has been hypothesized that the Dillon reservoir may be a noteworthy source of NTM among these watersheds as it provides the water for the Denver metropolitan area, a high NTM disease risk region of Colorado [19].

Among the three different types of samples studied, ice (67%) > tap water (33%) > plumbing biofilms (22%) for highest NTM positivity rate and species diversity recovered (Table 1). *M. chelonae* was commonly identified from ice sampled in this study (Fig. 3A). Despite ice water and ice machines as known niches for NTM in the hospital setting responsible for outbreaks that result in disease [33, 34], routine cleaning is often overlooked and could pose a source for healthcare associated NTM infections.

*M. abscessus* healthcare associated transmission has been investigated in Colorado and is likely rare and more likely to occur due to patients residing in the same Middle South Platte Cherry Creek watershed [20]. To our knowledge, this work is the first report of non-hospital associated environmental NTM sampling in Colorado. A novel aspect was applying MinION WGS to understand how environmental *M. chelonae* isolates that were recovered from varying environments genomically differed. We commonly detected genes involving molybdenum import which aligns with the relationship between molybdenum, Colorado, and NTM LD [21]. *M. chelonae* was recovered from all three risk regions, but more frequently identified among samples collected from the moderate risk area of Dillion.

Overall, drug sensitivity testing is a hallmark of clinical isolate testing, but less is known about the drug resistance genes of environmental NTM isolates. In general, *Mycobacteria* are intrinsically resistant to many different antibiotics, and this applies to *M. chelonae* [35, 36]. Brown-Elliott et al. recently identified a novel plasmid-mediated *erm* gene, *erm*(55), among clinical isolates of *M. chelonae* [32]. While we did not find the *erm*(55) gene among the *M. chelonae* isolates in this study, resistance genes for bicyclomycin (*bcr*), bleomycin (*ble*), daunorubicin/doxorubicin (*drrA*), tetracycline (*tetA*), and beta-lactams (*ampC*) were found. Bicyclomycin is a broad-spectrum antibiotic that selectively inhibits the transcription termination factor Rho [37]. While what is known about Rho function in NTM is limited, Rho has been shown to modulate the termination of RNA synthesis for both protein-coding and stable RNA genes in *M. tuberculosis* and enhances transcription [38]. Bleomycin is typically an anti-cancer drug that causes severe pulmonary toxicity, precluding its use as an antibacterial agent [39]. Daunorubicin/doxorubicin are two antibiotics used as cancer chemotherapy and inhibit DNA topoisomerase II [40] but have no known effect on mycobacteria. Tetracycline use has declined due to increased drug resistance, having been replaced by the glycylcyclines and other drugs against *M. chelonae* [41]. The beta-lactam combinations have been shown to work synergistically against clinical isolates of *M. abscessus* [42]. The finding of the *ampC* gene in *M. chelonae* is unexpected and justifies follow up studies to understand its contribution to *M. chelonae*.

Unlike the two reference *M. chelonae* genomes which were both collected from animal sources, the six sequenced environmental *M. chelonae* isolates contained genes involved in terpene metabolism (*linC*). Terpenes, *i.e.,* isoprenoids, are the largest class of small molecule producing natural products on earth as plant and fungi metabolites. However, without more study, the role of *linC* remains ill-defined. We also found *adhD* genes, related to acidic pH tolerance. Under low pH conditions, *adhD* helps produce mycolic acid and maintain proper cell envelope permeability [43]. In *M. tuberculosis, adhD* is essential for survival within the acidic macrophage phagosome [44], although it is unclear if this gene also helps *M. chelonae* tolerate acidic conditions including acidic soil.

We compared the five Colorado environmental *M. chelonae* isolates to the one Hawai’i *M. chelonae* and found the two Colorado hospital isolates and the Hawai’i isolate each contained *dpnA*, but only the Hawai’i *M. chelonae* had *dpnM*. Both *dpnA/M* genes encode methyltransferases that prevent cleavage by the Dpn II restriction enzyme, but while DpnM can only methylate double stranded DNA, DpnA can methylate both single and double stranded DNA [45]. The *dpnM* gene function appears redundant when in the presence of *dpnA* which may explain its absence from the Colorado isolates. Also, while all six isolates harbored copies of ligase genes, *baiA* and *baiE*, only the five Colorado isolates had *baiB*, a ligase important for bile acid 7 alpha-dehydroxylation. The other ligase pathway genes [46] were not identified in any of the isolates so, this set of genes may perform a different function or be remnants of a lost pathway. Study of additional *M. chelonae* isolates from both states and other geographic locations will be required to determine if the presence and absence of these genes occurred randomly or reflect a true difference due to geography, possibly arising from the difference in climate, soil acidity, or other factors.

Cushman et al*.* reported increased expression of intrinsic antibiotic resistance genes in *M. chelonae* carrying the prophages BPs and McProf, which conferred resistance to amikacin [47]. We did not find evidence of BPs or McProf in our sequenced isolates. Their isolates with phage also showed increased expression of *whiB7*, a mycobacterial virulence gene that contributes to amikacin and clarithromycin resistance [48]. Our findings of *M. chelonae* prophages align with others who report their frequent detection among NTM [49, 50] and should be considered areas of future therapeutic development.

There are limitations to this work. First, the sample size of this study was small, and a larger sampling study should be performed to include more sampling sites within risk areas with longitudinal data collection to understand how NTM diversity may change over time. Without the genomic analyses of many more isolates, we are unable to make generalizable conclusions regarding biological differences among *M. chelonae* isolates. Second, environmental sampling was only performed at publicly accessible sites and did not include natural areas or patient and non-patient households. A third limitation was the inability to perform WGS on the Illumina sequencing platform for the Colorado *M. chelonae* isolates. The addition of short read sequencing to MinION long read data could improve assembly accuracy and contiguity.

## Conclusions

MinION whole genome sequencing was performed on six *M. chelonae* isolates to understand the genetic differences among isolates recovered from a variety of environmental sources. The Colorado isolates were genetically similar to the Hawai’i isolate and *M. chelonae* ATCC19237 demonstrating the universality of *M. chelonae*. Each environmental isolate harbored drug resistance genes suggesting that these opportunistic pathogens already contain some of the tools to evade common antibiotic treatments. Future research could include analyses of how these genomic differences compare to a large collection of clinical isolates and affect the prevalence of NTM LD by geographic locations. An expansion of the quantity of environmental samples in Colorado would allow for a better representation of the abundance and species diversity of NTM in areas of modest LD risk, allowing for a stronger comparison to the large quantity of environmental samples collected from geographic areas of high LD burden.

### Supplementary Information

Below is the link to the electronic supplementary material.

Supplementary file1 (PDF 714 KB)
