# Supplementary material for: Common Features of Environmental Mycobacterium chelonae from Colorado Using Partial and Whole Genomic Sequence Analyses
Source: Curr Microbiol. 2024 Jan 18;81(2):69. doi: 10.1007/s00284-023-03589-2 (PMC10796651; doi:10.1007/s00284-023-03589-2)
Supplement: Supplementary file 1 — Supplementary file1 (PDF 714 KB) [file 284_2023_3589_MOESM1_ESM.pdf]

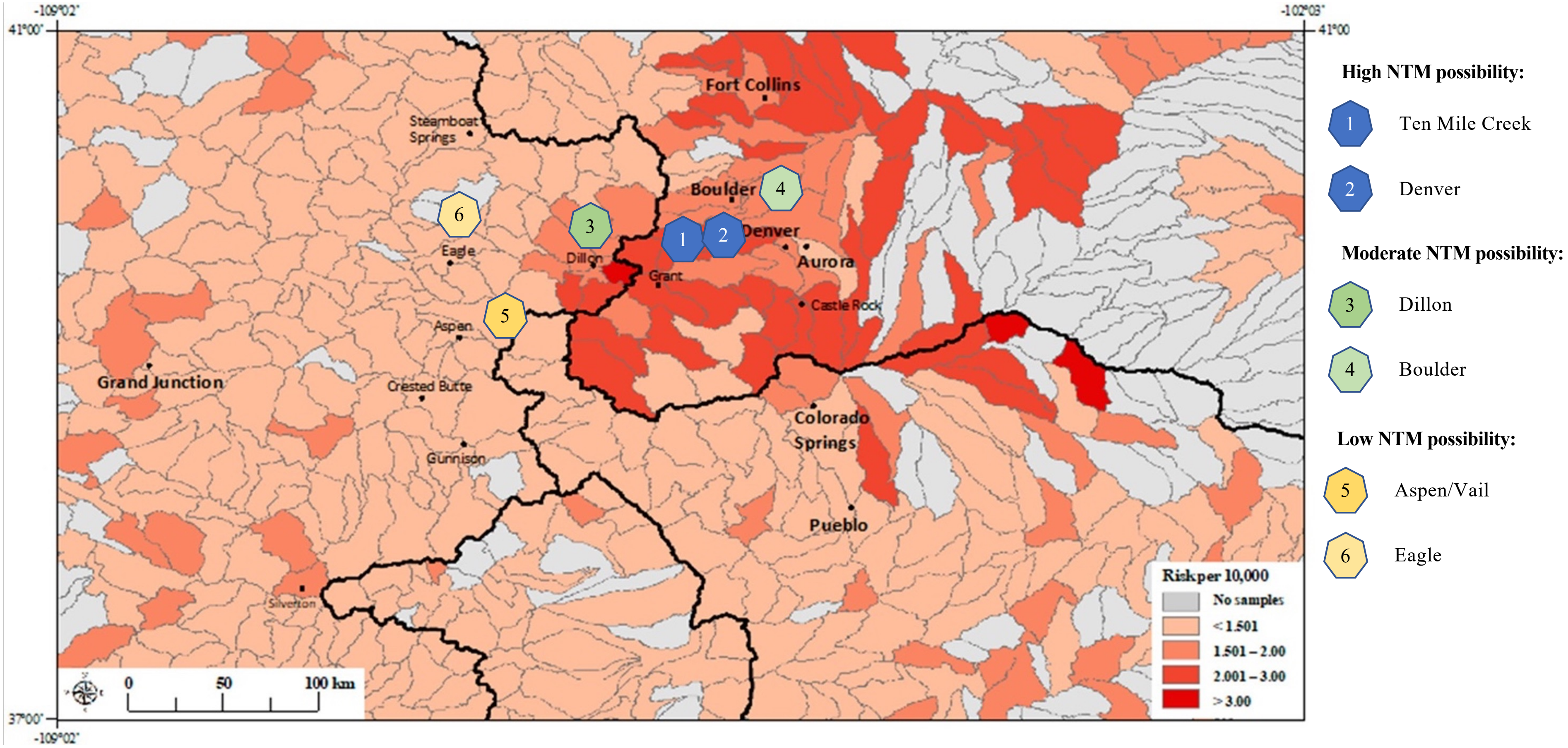

Online Resource 1. Fitted NTM lung disease risk estimates per watershed shown in map based on work by Lipner *et al.,* 2020. Shown with permission from co-author Lipner. A total of 54 environmental samples were collected = (3 risk areas) x (2 sites per area) x (3 sampling locations per site) x (3 different types of samples were collected).
